# Playing the field: The molecular basis of fruit morphology-based bet-hedging

**DOI:** 10.1093/plcell/koae119

**Published:** 2024-04-15

**Authors:** Leonard Blaschek

**Affiliations:** Assistant Features Editor, The Plant Cell, American Society of Plant Biologists; Department of Plant & Environmental Sciences, University of Copenhagen, 1871 Frederiksberg C, Denmark

Extreme weather fluctuations, as well as broader changes in climate, can severely endanger the survival of germinating seedlings. To prevent the total loss of offspring in such scenarios, some plant species hedge their bets by creating multiple types of seed, each with distinct germination behaviors. Some of these seeds will germinate with the first occurrence of favorable conditions—for example, the first rainfall or the beginning of spring. Other seeds remain dormant, forming soil-based seed banks that will gradually germinate over subsequent seasons or years. While such strategies diminish the number of offspring in an ideal environment, they ensure the survival of the population through extreme conditions. This “bet-hedging” can extend to the morphology of the diaspore, which is the seed (or spore) plus tissues, such as the fruit pericarp, that aid seed dispersal. Diaspore polymorphism has independently evolved several times and can confer ecophysiological advantages in unpredictable environments and climates. Crop domestication, on the other hand, actively selected against stochastic seed dormancy—especially as related to diaspore polymorphisms—to increase predictability and yield in controlled environments (Nave et al. 2016). This left us with no economically important crop or model species to investigate the molecular mechanisms behind these phenomena.

In this issue, **Jake Chandler and colleagues (Chandler et al. 2024)** report a detailed transcriptomic and metabolomic investigation into the mechanistic basis of diaspore dimorphism in *Aethionema arabicum*. A member of the Brassicaceae adapted to arid environments, *Ae. arabicum* produces 2 diaspore morphs: dehiscent fruits, which shed 4 to 6 mucilaginous seeds, and indehiscent fruits enclosing a single, nonmucilaginous seed (Lenser et al. 2016). The indehiscent fruits delay seed germination and promote spatial dispersal, increasing the likelihood of germination in a permissive environment (Arshad et al. 2019). The ratio of dehiscent to indehiscent fruits increases when the parental plants are exposed to higher temperatures after pollination. The extent of seed dormancy is further affected by imbibition temperature and—for the indehiscent fruits—the presence of the pericarp. To disentangle these effects, the authors analyzed seeds enclosed by the pericarp in the indehiscent fruits, seeds manually excised from the pericarp in indehiscent fruits, and seeds from dehiscent fruits (Fig.). Seeds were taken from parental plants grown at 20°C or 25°C and imbibed between 9°C and 24°C. Dehiscent fruits from parental plants grown at 25°C generally produced seeds with near-complete germination. Seeds excised from indehiscent fruits germinated more slowly, but deep dormancy was only observed when seeds were left enclosed in the indehiscent pericarp. Hence, the extent of seed dormancy was fine-tuned by temperature-sensitive mechanisms during both seed-setting and imbibition. Detailed transcriptomic analyses of the diaspore morphs under varying growth and imbibition regimes facilitated the high-confidence clustering of gene expression according to their behavior in dormant and germinating seeds. The resulting modules represented various degrees of gene coexpression between the different samples and regimes, providing a rich resource for further study that is readily accessible at plantcode.cup.uni-freiburg.de/aetar_db/.

**Figure.**
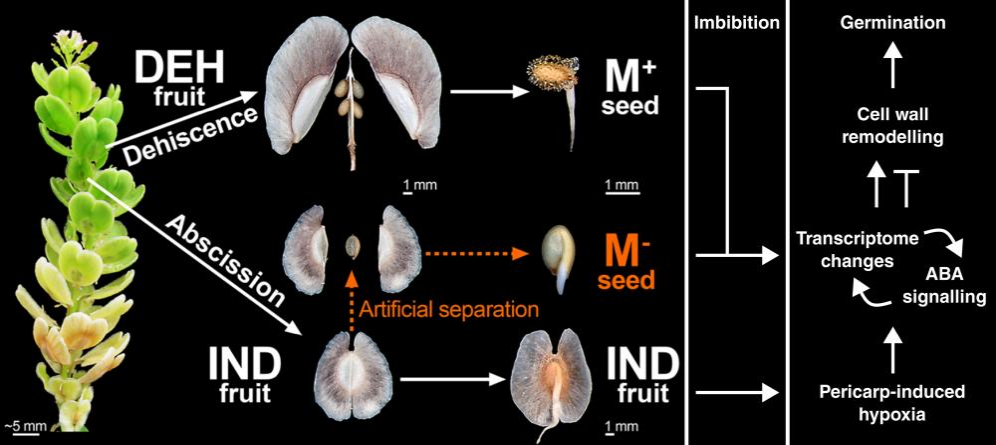
Diaspore dimorphism in *Ae. arabicum* and the basic mechanism delaying germination of seeds within indehiscent fruits. M^+^, mucilaginous seed from dehiscent (DEH) fruit; M^−^, nonmucilaginous seed excised from indehiscent (IND) fruit. Adapted from Chandler *et al*. (2024), Figure 1.

In agreement with their different germination efficiency, the transcriptome of excised seeds from indehiscent fruits was distinct from that of seeds still enclosed in the fruit. Previous reports from species with monomorphic diaspores showed roles for both ABA accumulation/sensitivity (Abley et al. 2021) and pericarp-induced hypoxia (Mendiondo et al. 2010) in seed dormancy. Investigating the contribution of these mechanisms in *Ae. arabicum* showed that seeds excised from indehiscent fruits treated with ABA fully mimicked the dormancy exhibited by seeds enclosed in the indehiscent fruit. This aligns with reports from Arabidopsis, where the balance between ABA and gibberellic acid explains observed variations in seed dormancy (Abley et al. 2021). The ABA treatment did not, however, mimic the coinciding transcriptomic effects of pericarp enclosure. Hypoxia during the imbibition of the excised seed, on the other hand, mimicked both the germination and transcriptome behavior of seeds within indehiscent fruits. Together, these observations suggest either that hypoxia acts upstream of ABA signaling or that there are at least 2, partly independent pathways that suppress germination in response to ABA and/or hypoxia in *Ae. arabicum*.

The exact interplay between ABA response and hypoxia awaits unraveling. To that end, future work will have to carefully distinguish transcriptional from post-translational responses to identify potential feedback or feed-forward loops. The detailed transcriptomic landscape described by Chandler et al. (2024) also invites further analysis to identify candidate genes and facilitate hypothesis-driven follow-up work. Altogether, the authors have made substantial advances toward understanding the molecular basis of diaspore dimorphism in the process of establishing *Ae. arabicum* as a powerful model organism.

## References

[koae119-B1] Abley K , Formosa-Jordan P, Tavares H, Chan EY, Afsharinafar M, Leyser O, Locke JC. An ABA-GA bistable switch can account for natural variation in the variability of arabidopsis seed germination time. Elife. 2021:10:e59485. 10.7554/eLife.59485 34059197 PMC8169117

[koae119-B2] Arshad W , Sperber K, Steinbrecher T, Nichols B, Jansen VAA, Leubner-Metzger G, Mummenhoff K. Dispersal biophysics and adaptive significance of dimorphic diaspores in the annual *Aethionema arabicum* (Brassicaceae). New Phytol. 2019:221(3):1434–1446. 10.1111/nph.15490 30230555 PMC6492137

[koae119-B3] Chandler JO , Wilhelmsson PKI, Fernandez-Pozo N, Graeber K, Arshad W, Pérez M, Steinbrecher T, Ullrich KK, Nguyen T-P, Mérai Z, et al The dimorphic diaspore model *Aethionema Arabicum* (Brassicaceae): distinct molecular and morphological control of responses to parental and germination temperatures. Plant Cell. 2024:36(7):2465–2490. 10.1093/plcell/koae085 PMC11218780 38513609

[koae119-B4] Lenser T , Graeber K, Cevik ÖS, Adigüzel N, Dönmez AA, Grosche C, Kettermann M, Mayland-Quellhorst S, Mérai Z, Mohammadin S, et al Developmental control and plasticity of fruit and seed dimorphism in *Aethionema arabicum*. Plant Physiol. 2016:172(3):1691–1707. 10.1104/pp.16.00838 27702842 PMC5100781

[koae119-B5] Mendiondo GM , Leymarie J, Farrant JM, Corbineau F, Benech-Arnold RL. Differential expression of abscisic acid metabolism and signalling genes induced by seed-covering structures or hypoxia in barley (*Hordeum vulgare* L.) grains. Seed Sci Res. 2010:20(2):69–77. 10.1017/S0960258509990262

[koae119-B6] Nave M , Avni R, Ben-Zvi B, Hale I, Distelfeld A. QTLs for uniform grain dimensions and germination selected during wheat domestication are co-located on chromosome 4B. Theor Appl Genet. 2016:129(7):1303–1315. 10.1007/s00122-016-2704-4 26993485

